# Effectiveness of a Collaborative, Virtual Outreach Curriculum for 4th-Year EM-bound Students at a Medical School Affiliated with a Historically Black College and University

**DOI:** 10.5811/westjem.18748

**Published:** 2024-12-16

**Authors:** Cortlyn Brown, Richard Carter, Nicholas Hartman, Aaryn Hammond, Emily MacNeill, Lynne Holden, Ava Pierce, Linelle Campbell, Marquita Norman

**Affiliations:** * Atrium Health Carolinas Medical Center, Department of Emergency Medicine, Charlotte, North Carolina; † Howard University, College of Medicine, Washington, DC; ‡ Wake Forest University, School of Medicine, Winston-Salem, North Carolina; § Albert Einstein College of Medicine, Montefiore Medical Center, Bronx, New York; ∥ UT Southwestern Medical Center, Dallas, Texas

## Abstract

**Background:**

Diversity within the physician workforce is associated with improved clinical outcomes and patient satisfaction. Despite this, the US physician workforce, particularly in emergency medicine (EM), remains relatively homogeneous. Of all Black medical school students in the US, 14% attend the four Historically Black Colleges and Universities (HBCU) that have a medical school. Unfortunately, none of these schools are affiliated with an academic EM program. Because of this, there is less professional mentorship focused on obtaining a career in EM and potentially less formal curricula for senior medical students doing their home sub-internship in EM.

**Objectives:**

Our objective was to fill the gap left by the absence of an academic EM department at Howard University College of Medicine (HUCOM) by creating a collaborative educational experience for fourth-year medical students during their home EM sub-internship. The curricular objectives were to teach core principles of EM, build relationships with students, and prepare them for pursuing EM residency training.

**Curricular Design:**

Four EM academic departments collaborated to create and implement a virtual curriculum using the six-step approach to curricular development.

**Impact/Effectiveness:**

After completion of the course, five students (100%) reported strongly agreeing with the following statements. These sessions 1) helped me learn the approach to core EM topics more than I would have been able to do on my own; 2) helped me learn key skills for excelling in an EM rotation more than I would have been able to do on my own; and 3) allowed me to connect with faculty and resident mentors to learn more about the field of EM. Of these five students, 80% and 20% reported strongly agreeing and agreeing, respectively, that these sessions helped them learn about the process of applying to and selecting an EM residency program.

## INTRODUCTION

### Need for Innovation

Medical students interested in emergency medicine (EM) who attend a historically Black college or university (HBCU) do not have the teaching and mentorship that occurs when a medical school is affiliated with an academic EM program. We formed a collaborative program among four academic EM departments to help fill this need for EM-bound students at Howard University College of Medicine (HUCOM). To our knowledge, this is the first such program to be reported in the literature.

### Background

A diverse physician workforce is associated with increased access to and utilization of the healthcare system, improved health outcomes and patient experience, and improved fiscal margins for hospitals.^1^ ^–^ ^4^ ^,^ ^4^ ^–^ ^6^ Despite this, the medical field as a whole has made minimal advances in increasing physician diversity. In 2008 the percentage of Black or Hispanic US physicians from all specialties was 6.3% and 5.5%, respectively. By 2018, however, those percentages were only 5.0% and 5.8%, respectively. Even more concerning given the diverse patient population that the emergency department (ED) serves, EM remains among the medical specialties with the lowest number of physicians from backgrounds under-represented in medicine (URiM). Between 2008–2018, the percentage of emergency physicians who identified as Black decreased from 5.0% to 4.5%, and stayed constant at 5.3% for Hispanic/Latinos.^7^

When surveyed, 35% of EM program directors reported that the small number of URiM residency applicants was the greatest barrier to obtaining a diverse residency class.^8^ Of all Black medical school students in the US, 14% attend four HBCUs with a medical school. Because none of these schools are affiliated with an academic EM program, their medical students have decreased exposure to EM in the pre-clinical years, less professional mentorship focused on obtaining a career in EM, and fewer formal curricula for senior medical students doing their home sub-internship (sub-I) in EM. This lack of mentorship has been identified as a critical barrier for URiM students across various specialties, contributing to lower application rates and residency placement. Studies suggest that mentorship increases both career satisfaction and inclusivity and the likelihood of these students entering and succeeding in competitive fields like EM.^9^ ^,^ ^10^ In addition, a national survey of clerkship directors found that having a structured, standardized sub-I curriculum significantly improved the preparedness of students for residency, especially when these rotations were affiliated with residency programs.^11^

The Emory University Department of Emergency Medicine created a program with Morehouse School of Medicine to provide guidance to medical students interested in EM. A total of 115 Morehouse students completed an EM clerkship at Emory, and 62.6% successfully matched into EM.^12^ While this program was successful, students typically rely on their home sub-I to prepare for mandatory away rotations. This absence of support from an academic department prior to away rotations may cause the students to find themselves less prepared and at a competitive disadvantage when they begin their away rotations. Furthermore, many EM residencies are not in proximity to a HBCU, requiring students to bear the financial burden of traveling to other cities and states for their away rotations.

At HUCOM, the EM sub-I relied heavily on an older, recorded online lecture series from an external institution, supplemented by bedside teaching from community attendings at one site, Howard Hospital. Students noted that the absence of formal educational components, such as weekly didactics, journal clubs, and simulation, resulted in limited exposure to “cutting-edge” EM practices. Moreover, the lack of interaction with academic attendings who are dedicated to medical student education, along with the absence of residents—who represent the next step in career progression—left students without access to critical mentorship and guidance. This gap hindered students’ ability to visualize their own progression and receive practical advice from individuals at a similar stage in training, further limiting their connection to the broader EM community.

To help overcome that barrier, we created a collaboration between four academic EDs and HUCOM in an attempt to augment curricular offerings for EM-interested students on their HUCOM fourth-year EM home rotation. The collaboration between four academic EDs broadens the exposure students receive to different teaching styles, institutional cultures, and clinical perspectives. This variety provides a more comprehensive educational experience than what can be offered by a single institution alone.

### Objective of Innovation

We aimed to address the absence of an academic ED at HUCOM by developing a collaborative educational experience. This program focuses on core principles of EM and residency preparation and was designed specifically for fourth-year medical students during their home EM sub-I at HUCOM. We obtained institutional board review approval from Wake Forest University School of Medicine.

### Development Process

We used the six-step approach to curricular development. All final curricular design and content was agreed upon by the faculty representatives at each of the four participating residency sites.^13^ ^,^ ^14^ 1) *Problem identification and general needs assessment.* Unlike traditional curriculum development where the need assessment is based on a specific health problem, our needs assessment was based on the need to increase the diversity of emergency clinicians by helping prepare under-represented students to succeed in away rotations and the match. 2) *Determining and prioritizing content.* While individuals at each participating institution were involved with teaching at their own institution, the needs of the HUCOM students were unique. Therefore, educational objectives were developed in conjunction with the faculty advisor to the fourth-year EM rotation at HUCOM who conducted stakeholder interviews with five current medical students and five alumni who had recently graduated and were currently in EM residencies across the country. It was decided that curricular content would include a mix of core EM topics (as determined from stakeholder interviews) and advising sessions.

After all sessions, students were provided with the contact information for the faculty lecturers and were encouraged to reach out. 3) *Goals and objectives*. Broad curricular goals were developed. These were to a) teach the approach to core complaints in EM; b) teach key skills in EM; c) demystify the process of applying to an EM residency program; and d) connect students with residents and faculty in the field of EM. After this, specific measurable lecture goals were developed based on cognitive, affective, and psychomotor objectives for the learner. 4) *Educational strategies*. We created an entirely virtual, four-week didactic program, with content organized into weekly four-hour blocks, each led by a different academic ED, on an interactive platform that allowed for case-based discussions, small-group discussions, and standard lecture format. Since implementation in 2022, the program has been mandatory for all students completing their fourth-year EM sub-I at HUCOM.

Each week, the sessions required the participation of four to five faculty members who volunteered their time, with the majority of lectures delivered by a single faculty member. However, select sessions, such as the “Application and Interviewing Process,” were co-led by a dynamic team consisting of the assistant program director, program director, and chief residents, providing a well-rounded perspective and valuable insights for the participants. Content was mapped and coordinated, and pre-reading was assigned from the Academy for Diversity and Inclusion in Emergency Medicine webinar series “How to Be a Successful EM Applicant” and the Clerkship Directors in Emergency Medicine/Society of Academic Emergecy Medicine M4 curriculum. Each day included a mix of clinical topics and “advising” sessions (Table 1). 5) *Implementation.* Approval from the EM director was obtained, and the curricula was implemented. 6) *Evaluation and feedback*. After each block of content, evaluations for each individual session (including the presenter) were sent to participating students via REDCap (Research Electronic Data Capture, hosted at Howard University School of Medicine.

**Figure 1.**
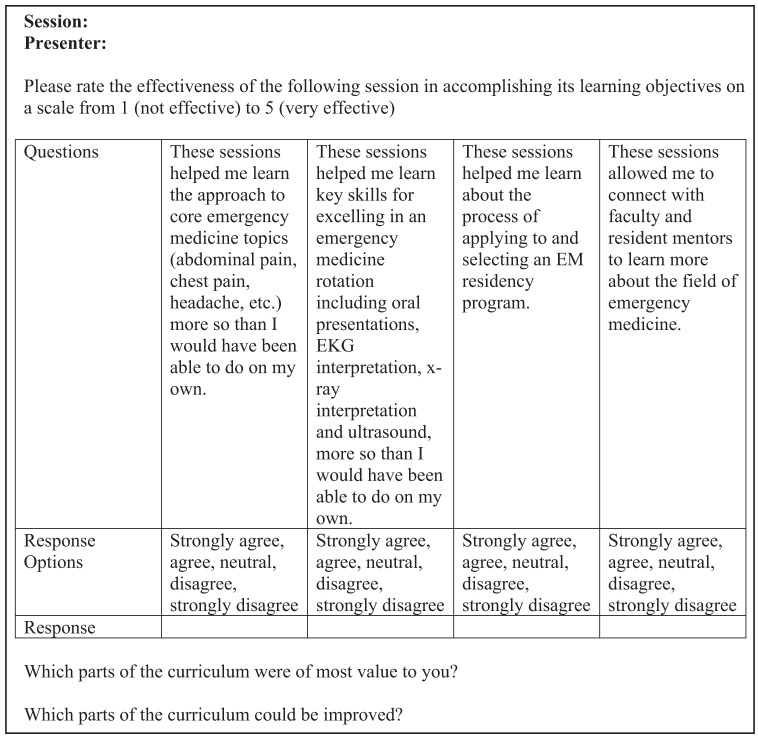
Evaluation form sent to students after each session.

These evaluations consisted of one question for each session: “Please rate the effectiveness of the following session in accomplishing its learning objectives: *Session, Presenter*.” At the end of the month-long program, an overall evaluation of the program was sent to participating students, also via RedCap. The program evaluation survey tool, including four multiple-choice questions regarding the overall learning objectives, is reflected in Figure 1. The tool also included two free-response questions: 1) “Which parts of the curriculum were of most value to you?”; and 2) “Which parts of the curriculum could be improved?” We refined the curricula each year during an end-of-year debrief.

**Table 1.** Curricula from sample block.

	Didactic session one Institution one	Didactic session two Institution two	Didactic session three Institution three	Didactic session four Institution four
Lecture topics	Personal statement	Presentation skills	How to choose the right program for you	Application and interviewing process
	Chest pain	Altered mental status	Toxicology overview	Headache
	Shortness of breath	Abdominal pain	Shock and sepsis	Gynecologic and urologic emergencies
	Radiographs	Electrocardiogram introduction	Vaginal bleeding	Endocrine and electrolytes
	Social emergency medicine	Ultrasound basics	Advanced trauma life support	Advanced cardiac life support, basic life support

### Implementation Phase

Prior to the first session, students were provided a spreadsheet with pre-session work, curriculum topics, presenting faculty and residents, dates and times, and links to access the weekly virtual sessions. Each EM program provided four hours of interactive didactics to the students according to the scheduled dates and times.

### Outcomes

A post-curricular survey found universal agreement from students that the curriculum was effective in meeting the above goals. Of the five students, 100% reported strongly agreeing with the following statements. These sessions 1) helped me learn the approach to core EM topics more than I would have been able to do on my own; 2) helped me learn key skills for excelling in an EM rotation more than I would have been able to do on my own; and 3) allowed me to connect with faculty and resident mentors to learn more about the field of EM. Of the five students, 80% and 20% reported strongly agreeing and agreeing, respectively, that these sessions helped them learn about the process of applying to and selecting an EM residency program.

Narrative feedback, such as the quotes below, from students highlighted the value of meeting with faculty and residents from different programs. from going through cases in real time.

Meeting the faculty and program directors at various EM programs really was the highlight of the curriculum. It was great to get an inside look at each program and learn more about their culture, approach, and the people there.

I really enjoyed hearing the residents’ perspective on how to navigate the application process.

Narrative feedback, such as the quotes below, also emphasized the value of the curriculum’s interactive nature and how traditionally in-person topics were effectively adapted for virtual learning.

My favorite part was participating in real-time cases. Being involved as the case unfolded felt like hands-on practice.

It was incredible to have the mechanisms of ultrasound explained in such detail. Breaking it down to the basics really helped me understand ultrasound for the first time.

## REFLECTIONS AND LESSONS LEARNED

### Engagement of the Home Institution

Successful implementation required active engagement from HUCOM, specifically the clerkship director and administrative staff, who served as lead contacts. Control over rotation scheduling was essential to ensure all students were fully engaged in the sessions. In addition, as participating institutions used various online platforms to communicate and disseminate curricula materials, such as *Tintinalli’s Emergency Medicine*, with their students, it was necessary to have HUCOM manage a central communications- and video-conferencing platform that was accessible to all lecturing institutions and participating students.

### Engagement of Collaborating Institutions

Recruiting faculty and residents for each institution’s week was challenging, but having representatives with strong connections in medical education made a significant difference. These relationships allowed them to quickly and effectively recruit lecturers, leveraging their networks to secure individuals who were both willing and enthusiastic to participate. This highlights the value of having institutional leads with established ties to their educational infrastructure, streamlining the recruitment process.

### Collaborative Power

The success of this project involved a high degree of trust as many of the institutional representatives had not worked together. To develop this trust, we followed the framework of engaging, listening, framing, envisioning, and committing.^15^ The power of this program is truly in the collective rather than the individual. While students could learn about atrial fibrillation from one institution, the real learning occurs when they see the collaboration, get a sense of the scope of EM as a professional field, and are able to interact with varied institutions that have different approaches to teaching and the practice of medicine.

### Challenges with Small Student Cohorts

Unlike traditional EM rotations that attract students from across the country, our program had a small cohort comprised solely of HUCOM students, as there was no affiliated residency. This small group size meant that if one student missed a session due to interviews, illness, or other reasons, it noticeably impacted the learning environment, limiting group dynamics and peer-to-peer learning.

### Program Limitations and Adaptations

Virtual learning posed challenges for teaching interactive skills such as ultrasound. We addressed this by incorporating case-based learning with curated image libraries and real-time feedback. To further enhance the learning experience, future iterations should explore the integration of ultrasound simulation software to better mimic hands-on scenarios.

### Scalability and Expansion

Although initially designed for HUCOM students, this model could be expanded to other medical schools without academic EDs, especially those with a high proportion of URiM students. With the opening of additional HBCU medical schools, there is an even greater need for programs that increase access to EM education.

### Limitations

Study limitations include the small sample size as well as lack of a comparison group. Future analyses will address these limitations and include evaluation of match outcomes as well as other learner-centered targets such as performance in Standardized Letters of Evaluation or subsequent rotations and intern year performance.
